# Effectiveness of WeChat-assisted preoperative education to reduce perioperative anxiety in breast cancer patients: a prospective randomized controlled study protocol

**DOI:** 10.1186/s13063-024-08071-3

**Published:** 2024-04-03

**Authors:** Xiao Xiao, Yi-Ding Zuo, Shu-Yu Kuang, Chun-Yuan Liu, Kun-Shao, Heng Wang, Si-Yu Yan, Feng Yu, Yan Xu, Li Zhou, Chun-Ling Jiang

**Affiliations:** 1 Department of Anesthesiology, The Research Units of West China (2018RU012), West China Hospital, Sichuan University, Chinese Academy of Medical Sciences, Chengdu, 610041 China; 2 Chongqing Liangping District People’s Hospital, Chongqing, 400700 China; 3 Xinxiang Central Hospital, Xinxiang, 453000, China; 4 West China Xiamen Hospital of Sichuan University, 361000 Xiamen, China

**Keywords:** WeChat public platform, Breast Cancer Resection, Preoperative Anxiety, STAI

## Abstract

**Background:**

Breast cancer is the most prevalent cancer among women globally, and surgical procedures continue to be the primary treatment. However, over 50% of patients experience preoperative anxiety due to the unknown and fear associated with surgery. Although drug therapy is commonly used to address this anxiety, its side effects have led to a heated debate regarding its effectiveness. Consequently, non-pharmacological therapies, such as preoperative education, have emerged as an alternative approach to alleviate anxiety. WeChat, a widely popular social media platform, offers a public platform that can potentially be utilized for effective preoperative education. This study aims to evaluate the use of WeChat public platform as a tool for preoperative education in patients undergoing breast surgery.

**Methods:**

This is a prospective, randomized, and controlled trial will involve 392 adult women scheduled for breast cancer resection. Participants will be randomly assigned to either the WeChat education group or the regular group. In addition to regular preoperative visits, the WeChat education group will also watch science videos through the WeChat public platform. The regular group will only receive education from ward nurses during preoperative visits. The primary outcome measure will be the incidence of preoperative anxiety, defined by scores of the State Anxiety Inventory (SAI) exceeding 40 points. Secondary outcome measures include the incidence of severe anxiety (SAI > 44) on the day before surgery, incidence of anxiety 72 h after surgery, incidence of severe anxiety 72 h after surgery, NRS scores for pain at rest and during activity 24, 48, and 72 h after surgery, incidence of nausea and vomiting within 24 h after surgery, subjective sleep score at 1 week postoperatively, quality of life QoR-15 scores at 1 and 3 months postoperatively, incidence of chronic pain at 3 months postoperatively, bowel function recovery, length of hospital stay, and hospitalization expenses.

**Discussion:**

This is the first clinical trial to investigate the use of WeChat public platform for delivering preoperative education on perioperative anxiety in breast cancer patients. By utilizing the renowned WeChat public platform, our study aims to improve patient outcomes by providing video education that explains the disease, surgery, and anesthesia in a more accessible manner, thereby reducing the incidence of perioperative anxiety. If our hypothesis is confirmed, this non-pharmacological approach can be universally acknowledged as a cost-effective and practical method in clinical care. Its application can also be extended to other medical fields beyond breast cancer.

**Trial registration:**

ClinicalTrials.gov, NCT05291494. Registered on 29 December 2021.

**Supplementary Information:**

The online version contains supplementary material available at 10.1186/s13063-024-08071-3.

## Introduction

Breast cancer is the most common form of cancer among women [1] and ranks as the second leading cause of cancer-related deaths [2]. Statistics suggest that 13% of females will develop breast cancer during their lifetime [2]. Surgery remains a crucial component of breast cancer treatment for non-metastasized cases. However, while surgery removes the tumor, it often inflicts significant psychological distress on patients. Previous empirical evidence indicates that 51% of patients experience preoperative anxiety [2], which can lead to various negative consequences. Additionally, postoperative changes in appearance further contribute to patient distress. Research shows that the incidence of moderate to severe anxiety before surgery reaches 53.4% [3, 4]. A study conducted by Rosiek A and his team has linked preoperative anxiety to adverse effects on blood pressure, heart rate, neuroendocrine system, postoperative recovery, and increased dependence on analgesics [5]. Moreover, preoperative anxiety has the potential to induce or exacerbate acute and chronic pain after surgery, as well as cause sleep disorders [6–8], all of which hinder patients’ early recovery. While drug therapy is commonly used to address preoperative anxiety, its clinical application is limited due to side effects such as hypotension, bradycardia, slow breathing, nausea, vomiting, allergies, and even shock [9, 10]. Recent studies have also associated the use of anti-anxiety drugs with longer hospital stays and postoperative adverse events, including reoperation, infection, and cardiovascular and cerebrovascular events [11]. Consequently, non-pharmacological therapies, such as preoperative education, have emerged as viable option for anxiety relief, gaining significant attention [12–14]. Specifically, preoperative education manuals have demonstrated benefits in decreasing patient anxiety levels [15], as highlighted by the research of Kiyohara and Harkness [16, 17]. However, these methods have inherent limitations related to time, space, and patients’ educational backgrounds. Patients may struggle to recall information provided by ward nurses or face difficulties in achieving a complete understanding, particularly among older individuals. Moreover, accessibility to education is restricted by availability and convenience. Therefore, the development of education videos has gained importance. As early as 1989, Herrmann successfully utilized video for preoperative education in cardiac catheterization procedures to reduce anxiety levels [18]. However, time and space constraints still posed challenges. Hence, it is crucial to identify a more convenient, non-pharmacological therapy based on video to effectively reduce preoperative anxiety.

WeChat is a comprehensive application that integrates social media, instant messaging, mobile payment, and e-commerce functions. It is widely regarded as one of the most popular social media platforms globally, surpassing the combined login time of the top four social platforms in American platforms [19]. In China, WeChat has become an indispensable part of daily life and boasts over 1 billion monthly active users [19]. Surprisingly, more than one third of users spend more than 4 h on the platform each day. In 2017 alone, an average of 38 billion messages were sent through WeChat daily, with 68 million of them being videos [19]. Among these features, the WeChat public platform serves as a vital source of information across various industries, allowing the transmission of information to specific users and interactive channels [19]. Apart from its basic functionalities such as group communication through videos, articles, and other content, it also serves as a medium for all-round communication with users. Importantly, it provides users with convenient access to relevant information at any time and from anywhere. Thus, the WeChat public platform has gained public recognition as an effective means of communication. However, its effectiveness as a tool for preoperative education remains unexplored.

As mentioned above, perioperative anxiety is primarily driven by the uncertainty surrounding disease prognosis as well as fears related to surgery and anesthesia. In response to this issue, our plan involves creating videos focusing on breast cancer prognosis, surgical methods, anesthesia techniques, and other perioperative management knowledge. These videos will be made available to patients via the WeChat public platform, allowing them to access the information whenever and wherever they desire. We anticipate that these measures will contribute to alleviating perioperative anxiety and pain, improving the quality of sleep and life, and facilitating better early postoperative recovery.

## Methods

### Study design, approval, and registration

**Fig. 1:**
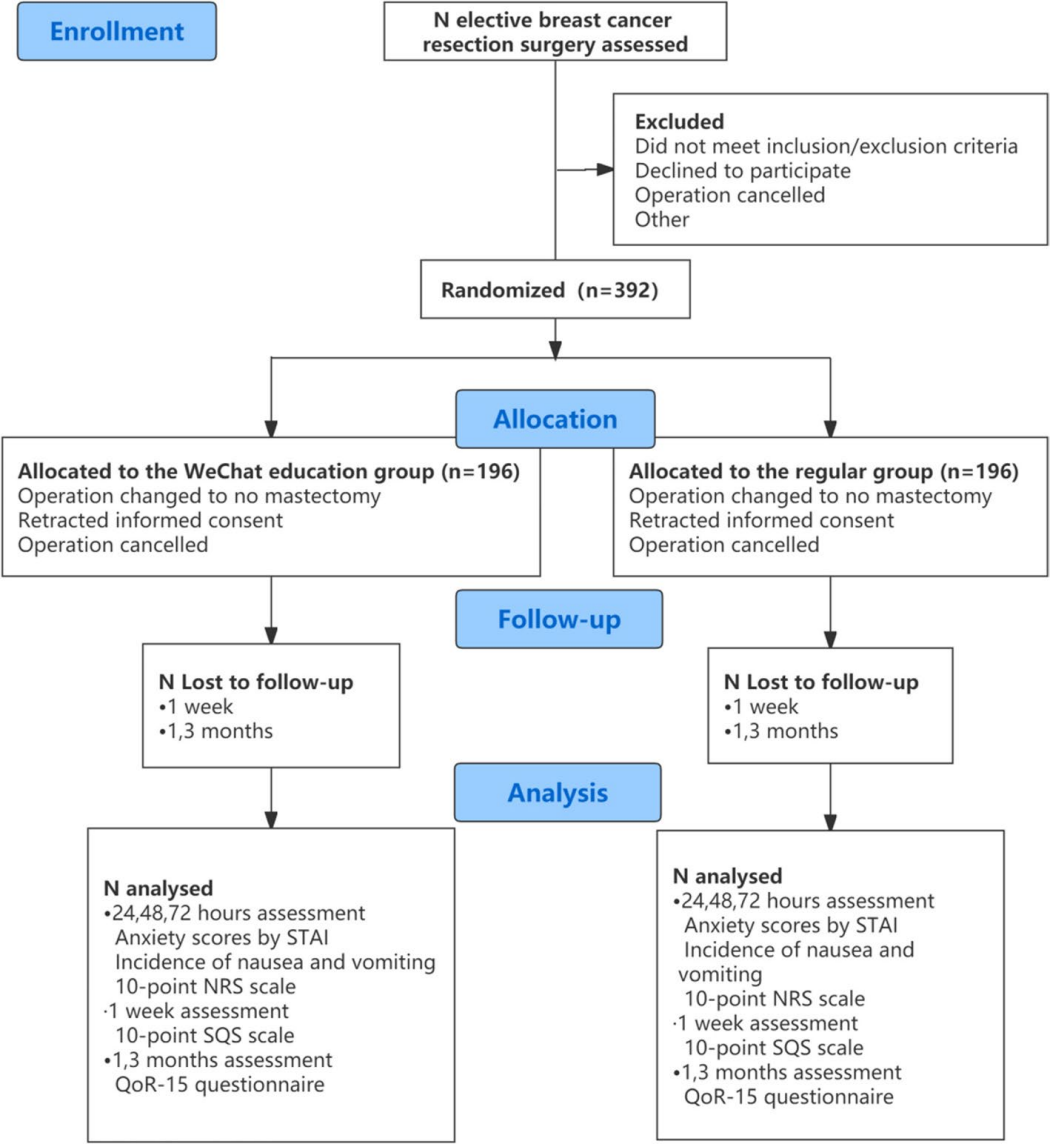
Consort diagram of study participant flow. STAI, state-trait anxiety inventory; NRS, numeric rating scale; SQS, sleep quality scale; QoR-15, 15-item Quality of Recovery Questionnaire

This prospective randomized controlled trial was conducted at West China Hospital of Sichuan University. The flowchart illustrating the test process can be found in Fig. 1. Patient recruitment began in February 2021. To ensure adherence to proper protocols, this trial adheres to the Standard Protocol Items: Recommendations for Interventional Trials (SPIRIT) 2013 [20, 21]. All registration, interventions, and evaluations are in accordance with the SPIRIT checklist [21]. The Ethics Committee of West China Hospital of Sichuan University has granted approval for this trial (2020–1138), and it has been registered in ClinicalTrials.gov. We have established a project steering committee, consisting of members from the anesthesiology department, breast surgery physicians, and nurses. Anesthesiology researchers are responsible for patient screening, assessment, perioperative management, and follow-up. Breast surgery physicians are in charge of determining the surgical approach and postoperative patient ward management. Breast surgery nurses are responsible for in-person perioperative education on the ward, and all of them are involved in formulating perioperative educational content. Furthermore, monthly group meetings will ensure the quality of patient inclusion. The project management group also will convene monthly meetings to review the progress of the trial. Additionally, the ethics committee will meet every 3 months to assess the conduct of the trial. Given that this is a low-risk intervention, a data monitoring committee is not necessary. There are no plans to collect, evaluate, or store biological specimens for genetic or molecular analysis in the current trial or for future ancillary studies.

### Study aim

The aim of our research is to reduce perioperative anxiety in patients diagnosed with breast cancer through preoperative education using the WeChat public platform. This intervention is expected to have a positive impact on early postoperative recovery, prognosis, and long-term life satisfaction.

### Participants

A total of 392 female patients scheduled for elective breast cancer resection at the West China Hospital (Sichuan, China) will be included in this study.

### Inclusion criteria

The inclusion criteria for this study are as follows: female patients undergoing elective breast cancer resection, aged 18 and 80 years, and classified as American Society of Anesthesiologists (ASA) physical status I–III.

### Exclusion criteria

Patients meeting one or more of the following criteria will be excluded from the study: those diagnosed with primary breast cancer combined with malignant tumors of other organs (e.g., lung, kidney, intestine, systemically metastasized breast cancer), patients with tumor recurrence after reoperation, and individuals unable to cooperate with the study due to communication disorders or other reasons.

### Randomization, allocation, and concealment

Upon admission to the hospital, all prospective participants will receive written consent. The consent form will request permission to use their data should they choose to withdraw from the trial as well as allow the research team to share relevant data with participating universities or regulatory authorities. Biological specimens will not be collected or stored as part of this trial. After obtaining informed consent, patients will be randomized into either the WeChat education group or the regular group at a 1:1 ratio. Randomization lists will be generated using the IBM SPSS statistics 23.0 software scheduler computer, and the lists will be concealed from screeners, assessors, and participants. Group allocations and detailed intervention plans will be prepared and sealed in opaque envelopes labeled with each participant’s serial number. These envelopes will be held by the screener responsible for participant screening. When a patient is recruited, the corresponding envelope will be sent to a researcher who will open it and implement the intervention according to the instructions provided (WeChat education or regular preoperative education). Another researcher, responsible for preoperative evaluation and postoperative follow-up, will remain unaware of the patients’ group assignments.

### Patient and public involvement

Researchers actively collaborate with members of the public before conducting the study, working together to plan preoperative educational materials, develop research protocols, and explain the research process and objectives. Throughout the study, researchers also assess the level of discomfort or difficulty associated with the intervention in consultation with the participants. Once the experiment is concluded, the research results are disseminated to the public through various online channels.

### Interventions

Patients who meet the inclusion criteria will be randomly assigned to either the WeChat preoperative education group or the regular group. Two researchers named Xiao Xiao and Yu Feng will respectively handle preoperative education, preoperative evaluation, and postoperative follow-up.

#### WeChat preoperative education team

In this group, patients are initially assessed by a researcher upon admission to evaluate their anxiety and sleep levels using the State-Trait Anxiety Inventory (STAI) scale and the Sleep Quality Scale (SQS). After the assessment, patients are instructed to follow the WeChat public platform and watch educational videos at their convenience. The content of these videos is collectively decided by the breast surgeon, anesthetists, and ward nurse, including the overall prognosis, surgical approach, postoperative rehabilitation exercises, preparation for anesthesia, anesthesia approach, and possible effects of anesthesia. The videos are presented in a clear and easily understandable manner, ensuring that participants of all ages and educational backgrounds can comprehend the information. Participants can simply click on the video dialogue box within the public platform to play and watch the videos without any restrictions.

#### Regular group

In this group, patients undergo the same anxiety and sleep assessment conducted by the same researcher during the admission process, utilizing the State-Trait Anxiety Inventory (STAI) scale and the SQS. Following the assessment, patients receive oral instruction from the ward nurse, covering the same content as the WeChat education videos (patients in the intervention group also receive oral instructions from the ward nurse).

### Perioperative management

Both groups underwent a routine pre-operative visit and a State-Trait Anxiety Inventory (STAI) test conducted by one researcher on the day before surgery. On the day of surgery, patients entered the operating room where they underwent monitoring of ECG, heart rate, non-invasive blood pressure, and nitrogen dioxide levels. Anesthesia was induced using midazolam 2 mg, propofol 1.5–2.5 mg/kg, sufentanil 0.3 µg/kg, and cis-atracurium 0.2 mg/kg and maintained intraoperatively with propofol, desflurane, or sevoflurane, along with continuous infusion of remifentanil 0.05–0.2 μg/kg/min. Intermittent infusions of sufentanil and cis-atracurium were also administered. At 72 h postoperatively, anxiety scores based on the STAI were evaluated in both groups by the same investigator. Acute pain scores based on the numeric rating scale (NRS) were assessed at 24, 48, and 72 h postoperatively. The use of analgesics and the incidence of nausea and vomiting at 24 h postoperatively were also recorded.

### Data collection

#### Baseline patient characteristics

Patient’s demographics, comorbidity, past surgical history, anxiety scores on admission, and sleep scores will be recorded.

#### Anxiety scores 1 day before and 72 h after surgery

Patients will be assessed for anxiety using the State-Trait Anxiety Inventory (STAI) scale [22, 23] 1 day before surgery and at 72 h after surgery. This 40-item scale is divided into two subscales, each with 20 items. The first part, the “State Anxiety Inventory (S-AI) scale,” evaluates the patient’s current state of anxiety and asks how the participant feels “right now.” The second part, the “Trait Anxiety Inventory (T-AI),” assesses the relatively stable tendency to be anxious [24]. Since this study focused on short-term changes in anxiety levels, the State Anxiety Inventory (S-AI) scale is selected. The total score ranges from 20 to 80, with higher scores indicating more severe anxiety. A score above 40 indicates clinical anxiety [24, 25], and a score exceeding 44 strongly suggests severe anxiety [26].

#### Acute pain assessment at 24, 48, and 72 h postoperatively

At 24, 48, and 72 h after surgery, patients will be evaluated for pain at rest and during activity (coughing or taking three deep breaths) using the NRS (0 = no pain, 10 = worst pain imaginable) [27].

#### Follow-up at 1 week, 1 month, and 3 months (Table 1)

Patients will be followed up via telephone at 1 week, 1 month, and 3 months after surgery. The SQS [28] and the 15-item quality of recovery score (QoR-15 questionnaire) [29] will be used to assess the sleep quality and overall life quality. Each participant will provide at least two telephone numbers to ensure proper follow-up and minimize loss of contact.

**Table 1:** Schedule of recruitment, interventions, and assessments

Timepoint	Study period								
	Enrollment	Allocation		Post allocation					
	-d	0	-d_0_	24h	48h	72h	W_1_	m_1_	m_3_
Enrollment									
Case diagnosis	X								
Inclusion criteria	X								
Exclusion criteria	X								
Informed consent	X								
Randomization	X								
Allocation		X							
Interventions									
WeChat education	X								
Videos and/or regular	X		X						
Preoperative visits	X								
Assessments									
Anxiety scores	X		X			X			
PONV	X			X					
Sleep scores	X						X		
Pain assessment	X			X	X	X			
Quality of life scores	X							X	X

According to SPIRIT 2013 statement: defining standard protocol items for clinical trials

*PONV*, postoperative nausea and vomiting

### Outcomes

#### Primary outcome

The primary outcome indicator is the incidence of preoperative anxiety, which is defined as a score of > 40 on the S-AI scale [24].

#### Secondary outcomes

The secondary outcome indicators include:

1. The incidence of severe anxiety (SAI > 44) 1 day before surgery;
2. The incidence of anxiety 72 h after surgery;
3. The incidence of severe anxiety 72 h after surgery;
4. NRS scores for pain at rest and during activity 24, 48, and 72 h after surgery (active status defined as 3 deep breaths or 1 cough);
5. The incidence of nausea and vomiting within 24 h after surgery;
6. Subjective sleep score (SQS) at 1 week postoperatively;
7. Quality of life QoR-15 scores at 1 and 3 months postoperatively.

### Adverse events and safety

Any adverse events, defined as any functional impairment caused by the intervention, will be recorded. Serious adverse events such as Internet/privacy issues targeting breast cancer patients and insurance issues will also be recorded. In the case of any adverse events, appropriate treatment, changes, or discontinuation of the intervention will be offered to the patient. Adverse events must be reported immediately to the principal investigator and the ethics committee to determine whether the patient needs to be withdrawn from the trial.

### Statistics

#### Sample size estimation

The sample size for this study was determined based on the results of our pre-test, which indicated a prevalence of preoperative anxiety in breast cancer patients of 57.1%. It was assumed that the intervention in this study would reduce the incidence of preoperative anxiety by 30%, with a test efficacy of 90% and an alpha of 0.05 (two-sided). The final sample size was determined to be 392 cases, considering a potential loss to follow-up or dropout rate of 10%.

#### Statistical analysis

Statistical analysis was performed using the software SPSS 23.0. The data will be presented as mean ± standard deviation or numbers (percentages). Baseline characteristics will be compared executing the chi-square test or Fisher’s exact test, Student’s *t*-test, or non-parametric tests. The primary outcomes (incidence of preoperative anxiety) will be compared using the chi-square test. Secondary outcomes, including the incidence of anxiety and severe anxiety at 72 h postoperatively, the incidence of nausea and vomiting at 24 h postoperatively, and the incidence of chronic pain at 3 months postoperatively, will be compared between groups using the chi-square test. NRS scores for pain at rest and during activity at 24, 48, and 72 h postoperatively, subjective sleep scores at 1 week postoperatively, quality of life QoR-15 scores at 1 month and 3 months postoperatively, length of hospital stay, and hospitalization costs will be compared using the *t*-test or rank sum test depending on their normality. Subgroup analysis will be conducted for female breast cancer patients who have pre-existing mental/psychological disorders and/or were using psychotropic medications in the final outcomes. A *p*-value < 0.05 will be considered statistically significant.

## Discussion

This is the first clinical trial that aims to deliver preoperative education on perioperative anxiety through the WeChat public platform specifically for breast cancer patients. The main objective of this randomized controlled trial is to reduce the incidence of perioperative anxiety in breast cancer patients, ultimately leading to improved early postoperative recovery and patient prognosis.

Perioperative anxiety plays a crucial role in both the prognosis and postoperative recovery of patients. Research has consistently shown that a significant proportion of patients (approximately 50–60%) who undergo mastectomy for breast cancer experience significant preoperative anxiety[4, 30]. Notably, pre-operative anxiety can have various negative effects, including hemodynamic changes, cardiac arrhythmias, increased pain level, and higher doses of anesthetics being required [31, 32]. Furthermore, it is associated with an increased risk of postoperative complications such as nausea, vomiting, and other serious complications [33], which ultimately impact the quality of postoperative recovery and overall patient well-being [34]. Therefore, it is imperative to explore effective approaches to reduce perioperative anxiety in this patient population.

Various non-surgical antianxiety therapies, such as video, music therapy [35], acupuncture [36], and hypnotherapy [37], have been studied extensively and have shown promise in reducing perioperative anxiety among breast cancer patients. However, despite their potential benefits, these therapies are not widely used in clinical practice due to the lack of standardized approaches. For example, music therapy [38] faces challenges in matching different types of music to specific age groups, making its implementation difficult. Similarly, acupuncture [39] lacks clarity regarding the optimal timing and frequency of administration perioperatively. Hypnotherapy [37, 40] also presents challenges as it requires highly skilled clinicians. Considering the limitations associated with these non-surgical antianxiety therapies, this study carefully selected video as a means of relieving perioperative anxiety in elective breast cancer patients. Video therapy is simple, easy to implement, and widely accessible. The study aims to investigate whether preoperative online education effectively reduces perioperative anxiety in patients undergoing elective breast surgery, with preoperative anxiety serving as the primary outcome indicator. The overall impact on perioperative anxiety and postoperative recovery will be evaluated.

Smartphones are wildly recognized as the most extensively used device for accessing the Internet on a daily basis, accounting for a surprising 98.7% [41] of usage. The primary purpose of accessing the Internet through smartphones is online communication, which includes sending or receiving text, voice picture, or video messages via various applications. Moreover, social media platform like WeChat have eliminated time and space restrictions, enabling real-time health behavior interventions by sending messages to individuals across different age groups. This mode of communication is more effective than traditional booklets and posters [42]. Additionally, this convenient form of communication allows individuals to receive support from peers, family members, and healthcare professionals. Research has shown that health interventions delivered through social media platforms have great potential in promoting health and health-related behaviors [42].

Our study aims to improve the prognosis of patients with breast cancer by utilizing the WeChat public platform. Through video education, we provide information on the disease, surgery, and anesthesia preoperatively to reduce perioperative anxiety. Even though the educational content is highly accessible, there may still be a very small subset of patients who might misinterpret the provided information, potentially leading to biases and/or misunderstandings. Researcher will make every effort to assess the extent of patients’ understanding of the educational content to minimize bias in the study. This approach has not been extensively studied in the field of perioperative rehabilitation. If our hypothesis is confirmed, this immediate, handy, and flexible non-pharmacological method could be widely recognized as a cost-effective and practical approach in clinical care. It is important to note that the choice of social media platforms may vary based on geographical regions and local preferences. Furthermore, the application of this approach can be extended to other diseases such as liver cancer, cerebral tumor, and cardiovascular and respiratory diseases. By creating disease-specific educational content, we can potentially improve patient outcomes. Implementing this education for all inpatients could accelerate postoperative recovery and enhance long-term prognosis. These therapeutic benefits have the potential to reduce anxiety, increase compliance with medical advice, and improve preoperative and postoperative rehabilitation exercises.

Previous studies on perioperative anxiety have primarily focused on immediate indicators such as postoperative nausea, vomiting, and acute pain [43, 44]. However, it is crucial to consider long-term indicators, including chronic pain and quality of life, which greatly impact patient’s prognosis. More than 50% of patients undergoing breast surgery experience postoperative pain due to nerve and tissue damage [45]. Additionally, appearance changes can lead to varying degrees of anxiety, further affecting the patient’s quality of life. Therefore, in this study, we will also evaluate sleep, acute postoperative pain, and overall life quality as indicators to assess the potential improvement in long-term prognosis through WeChat-assisted preoperative education.

In conclusion, our study innovatively utilizes the WeChat platform to provide preoperative education for breast cancer patients, aiming to reduce perioperative anxiety, improve sleep and life quality, alleviate acute postoperative pain, and ultimately enhance overall prognosis.

## Trial status

Patient recruitment for this trial commenced on February 1, 2021, and participants enrollment was completed on March 31, 2022. The trial protocol (protocol ID: 2020–1138) was registered with ClinicalTrials.gov on December 29, 2021, and our trial registration number is NCT05291494.

### Supplementary Information

**Supplementary Material 1.**
